# A case report of right bundle branch block and junctional beats during ablation at the right ventricle outflow tract: metallic occluder’s unanticipated effect

**DOI:** 10.1093/ehjcr/ytae054

**Published:** 2024-01-31

**Authors:** Sijia Pu, Huiyi Liu, Hai Deng, Yumei Xue, Weidong Lin

**Affiliations:** Guangdong Cardiovascular Institute, Guangdong Provincial People’s Hospital (Guangdong Academy of Medical Sciences), Southern Medical University, 106 Zhongshan Er Rd, Guangzhou 510080, China; School of Medicine, South China University of Technology, Guangzhou 510006, China; Guangdong Cardiovascular Institute, Guangdong Provincial People’s Hospital (Guangdong Academy of Medical Sciences), Southern Medical University, 106 Zhongshan Er Rd, Guangzhou 510080, China; Guangdong Cardiovascular Institute, Guangdong Provincial People’s Hospital (Guangdong Academy of Medical Sciences), Southern Medical University, 106 Zhongshan Er Rd, Guangzhou 510080, China; Guangdong Cardiovascular Institute, Guangdong Provincial People’s Hospital (Guangdong Academy of Medical Sciences), Southern Medical University, 106 Zhongshan Er Rd, Guangzhou 510080, China; School of Medicine, South China University of Technology, Guangzhou 510006, China; Guangdong Cardiovascular Institute, Guangdong Provincial People’s Hospital (Guangdong Academy of Medical Sciences), Southern Medical University, 106 Zhongshan Er Rd, Guangzhou 510080, China

**Keywords:** Right bundle branch block, Premature ventricular complexes, Radiofrequency catheter ablation, Peri-membranous ventricular septal defect, His–Purkinje system, Case report

## Abstract

**Background:**

Previously, ablation at the outflow tract was considered to be safe and rarely affected the His–Purkinje system due to their spatial distance. However, we have reported a case of right bundle branch block (RBBB) and junctional beats that were recorded during radiofrequency catheter ablation in a patient who had a history of peri-membranous ventricular septal defect (pmVSD) closure and the implantation of a metallic occluder.

**Case summary:**

A 16-year-old girl with a metallic occluder for peri-membranous ventricular septum defect underwent an ablation procedure for premature ventricular complexes. During the ablation at the right ventricular outflow tract (RVOT), RBBB and junctional beats were recorded. His bundle potentials and the high-frequency potential generated by electrical interference were observed when mapping the margin of the occluder. To ensure safety, we attempted ablation at the right coronary cusp in the left ventricular outflow tract, which eventually proved to be successful, presenting an alternative ablation strategy.

**Conclusion:**

This is a rare report of RBBB and junctional beats observed during ablation at RVOT in a patient with pmVSD and a metallic occluder. The observed damage to the His–Purkinje system may be attributed to uncontrolled radiofrequency energy heating up caused by the metallic device. This case emphasizes the importance of thorough electroanatomic and activation mapping prior to starting the ablation procedure, especially in complicated cases. Furthermore, it suggests that ablation at a relatively remote position is both feasible and relatively safe for patients with occluder devices.

**Learning points**

- Conduction system dysfunction is a serious complication of radiofrequency catheter ablation for arrhythmia treatment.
- The ablation of outflow tract premature ventricular complexes was previously considered to be relatively safe with little risk of conduction disturbances.
- This case report suggests that ablation at the outflow tract may cause damage to the His–Purkinje system in some patients with metallic implants and congenital structural anomalies of the heart.

## Introduction

With the widespread use of radiofrequency catheter ablation (RFCA) for premature ventricular complexes (PVCs), it is crucial to optimize ablation strategies for both safety and efficacy, especially in patients with anatomical variants or a history of cardiac procedures. In recent years, transcatheter device closure has become a common approach for peri-membranous ventricular septal defects (pmVSDs). While complications and conduction abnormalities associated with this procedure have been extensively studied,^1,2^ there is limited research on the impact of implanted metallic occluders on RFCA.

In this case report, we present a patient with PVC who experienced right bundle branch block (RBBB) and junctional beats during RFCA at the right ventricular outflow tract (RVOT). Notably, the patient had previously undergone transcatheter closure for pmVSD.

## Summary figure

**Figure:**
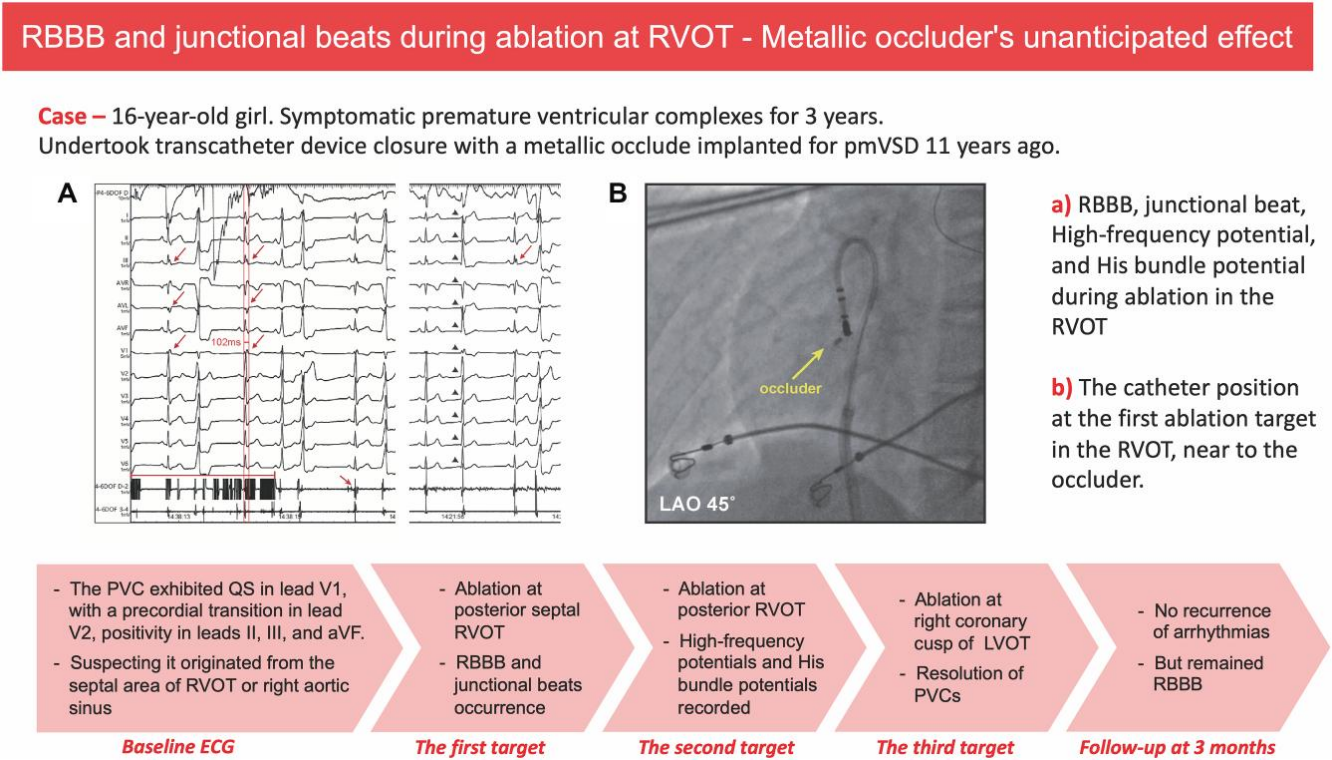


## Case presentation

A 16-year-old girl presented to our department with recurrent palpitations that had been occurring for 3 years. Eleven years prior, she had undergone transcatheter closure for pmVSD, and her post-operative electrocardiogram (ECG) showed sinus rhythm. Three years ago, she started experiencing palpitations and dizziness but never fainted. Recently, a 24-h Holter monitoring revealed a high burden of monomorphic PVCs, accounting for 17% of beats in 24 h. Transthoracic echocardiography showed a left ventricular ejection fraction of 62% and no residual septal shunt. Physical examination and laboratory tests were unremarkable.

**Figure 1:**
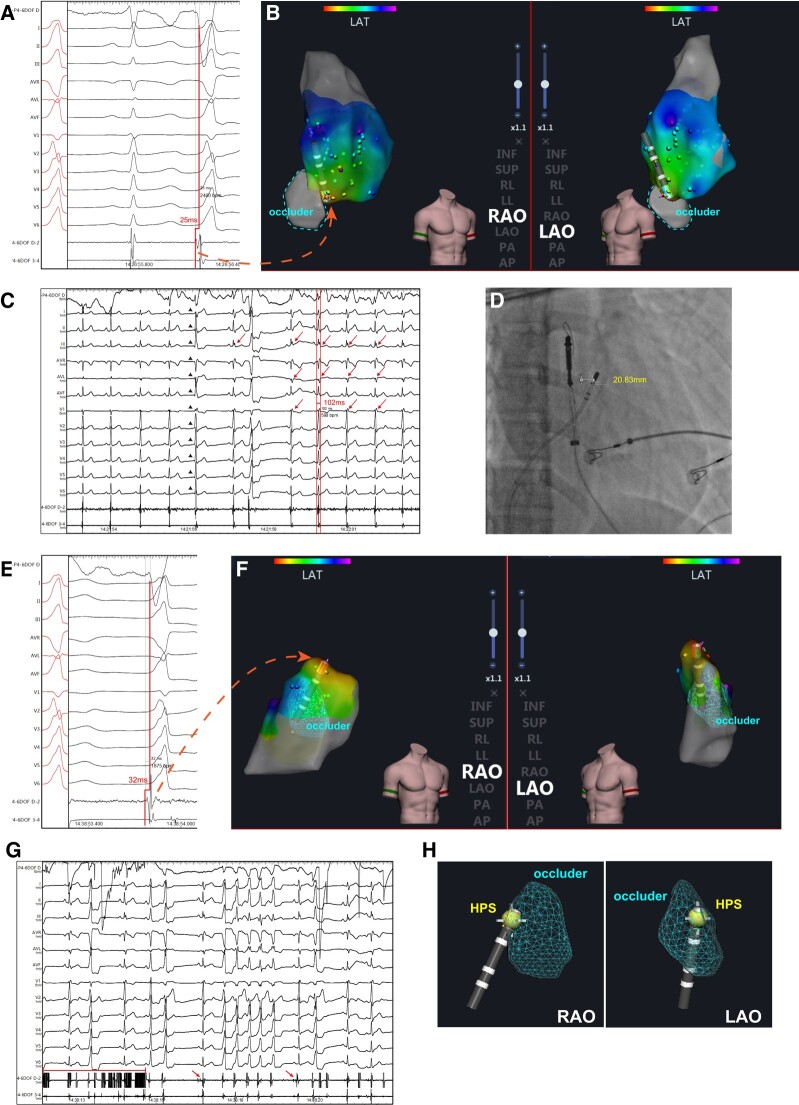
Electrophysiological study and radiofrequency catheter ablation in the right ventricular outflow tract. (*A*) Baseline and electrograms in right ventricular outflow tract on intracardiac electrocardiogram. (*B*) Three-dimensional electroanatomic mapping of local right ventricular outflow tract (arrow shows the earliest activation in this area); (*C*) right bundle branch block (arrows) and junctional beat (arrowheads) during radiofrequency catheter ablation at the first target; (*D*) the location of the ablation catheter and the occluder on fluoroscopy. (*E* and *F*) The earliest electrogram in right ventricular outflow tract on intracardiac electrocardiogram (*E*) and three-dimensional electroanatomic mapping (*F*), arrow shows the earliest activation. (*G*) High-frequency potential (transverse line) and His bundle potential (arrows) in right ventricular outflow tract. (*H*) Three-dimensional electroanatomic mapping of the occluder, the dot target represents the His bundle potential. HPS, His–Purkinje system; LAO, light anterior oblique; RAO, right anterior oblique.

After obtaining informed consent, an electrophysiological study was performed. The baseline ECG showed a PVC with a similar morphology to the documented one (*Figure 1A*). The PVC exhibited a QS morphology in lead V1, with a precordial transition in lead V2, positivity in leads II, III, and aVF, and no notch in the limb leads. We suspected that it was an idiopathic PVC originating from the septal area of RVOT or right aortic sinus.

Activation mapping was performed using an irrigated ablation catheter (OmniCool, APT Medical Inc, China) in conjunction with a three-dimensional (3D) electroanatomic mapping system (HT Viewer, APT Medical Inc, China). Initially, the local RVOT was mapped, specifically identifying the margin of the occluder through high-frequency potentials recorded near it in the 3D mapping. The area of interest was determined to be in the posterior septal RVOT, where a ventricular electrogram (EGM) preceded the QRS onset by 25 ms, indicating the proximity of the ablation catheter to the occluder (*Figures 1A* and *B* and *3B*). Ablation was conducted at 30 W in this location, resulting in the recording of a junctional beat and RBBB after 12 s of ablation (*Figure 1C*). The PVC morphology in lead V1 changed to rS, suggesting a more posterior origin. Subsequently, the RVOT beneath the pulmonary valve was remapped, revealing the earliest ventricular activity near the posterior septum with a timing of −32 ms (*Figure 1E* and *F*), again close to the occluder (*Figure 1D* and *F*). Due to the high-frequency potential generated by electric field interference, it was difficult to distinguish EGMs around the metallic occluder (*Figure 1G*). However, the His bundle (HB) potential was observed adjacent to the occluder (*Figure 1G* and *H*), indicating the presence of the conduction system in this region. During ablation at the location of the earliest activation for 9 s, junctional beats occurred again.

**Figure 2:**
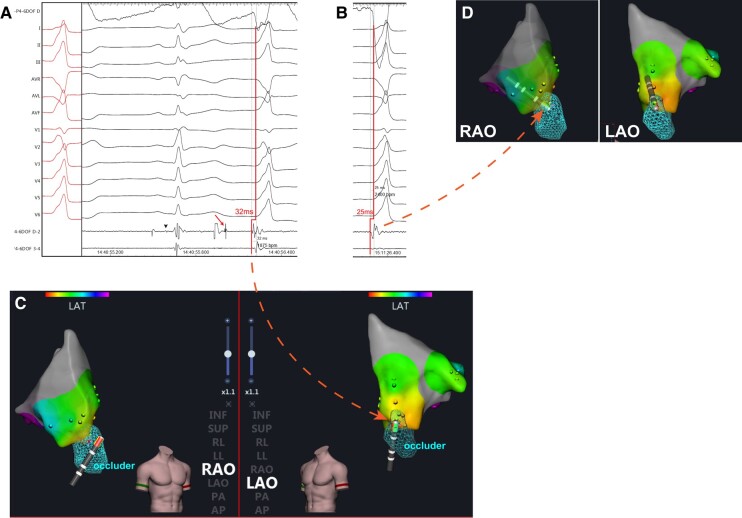
Electrophysiological study and radiofrequency catheter ablation in the left ventricular outflow tract. (*A* and *C*) The earliest electrogram in left ventricular outflow tract on intracardiac electrocardiogram (*A*) and three-dimensional electroanatomic mapping (*C*). The arrow in *A* shows the high-frequency potential, and the arrowhead shows the His bundle potential. The arrow from *A* to *C* shows the earliest activation. (*B* and *D*) The earliest electrogram above right coronary cusp on intracardiac electrocardiogram (*B*) and three-dimensional electroanatomic mapping (*D*). LAO, light anterior oblique; RAO, right anterior oblique.

**Figure 3:**
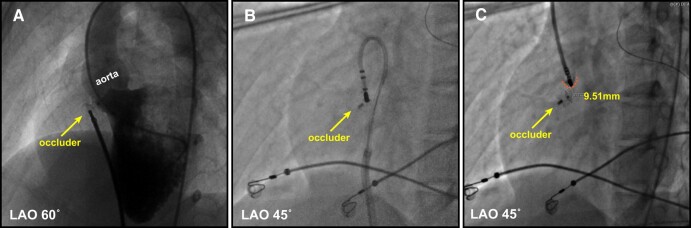
Fluoroscopic views of the occluder device and ablation catheter positions. (*A*) The view of the occluder device position when performing left ventricular angiography during a transcatheter device closure procedure 11 years ago. (*B*) The view of the catheter position at the first ablation target in the right ventricular outflow tract. (*C*) The view of the catheter position at the ablation target in the left ventricular outflow tract. Angiography was performed in the right coronary cusp. The distance between the ablation catheter and the occluder is 9.51 mm. LAO, light anterior oblique.

To ensure safety, we made the decision to guide the catheter to the corresponding anatomical position in the left ventricular outflow tract (LVOT) as the previous target. The earliest local EGM (*Figure 2A* and *C*) showed a timing of −32 ms compared with the QRS onset below the right coronary cusp (RCC), where both the HB potential and the high-frequency potential were still visible. Considering both safety and efficacy, we selected an ablation target above the RCC, 25 ms prior to the onset of QRS (*Figure 2B* and *D*). Confirmation through 3D mapping (*Figure 2D*) and angiography (*Figure 3C*) indicated that the catheter was positioned at the base of the RCC, with a distance of approximately 9.51 mm measured in fluoroscopy. Ablation at this location was successful, utilizing 30 W of power with a total radiofrequency delivery time of 90 s, and no further ventricular arrhythmia could be induced. The entire procedure lasted about one and a half hours, and the patient expressed satisfaction with the treatment. At the 3-month follow-up, the patient continued to display RBBB on Holter monitoring, with no further occurrences of PVCs.

## Discussion

Clearly, ablation strategies and target sites play a crucial role in the RFCA of PVCs in specific patients. To the best of our knowledge, there have been few reports on RFCA with a metallic occluder implanted in the membranous septum. The RBBB and junctional beats observed during ablation in this case suggest potential damage to the conduction system. Possible mechanisms include the complex anatomy of the His–Purkinje system (HPS), congenital conduction abnormalities, and uncontrolled radiofrequency energy heating by the metallic device.

It is crucial to have a thorough understanding of the complex local anatomy. In patients with pmVSD, the distribution of HPS is often inferior–posterior relative to the defect.^3^ Our previous research has shown that a distance less than 2 mm between the HPS and the defect is positively associated with post-procedural conduction abnormalities in transcatheter device closure for pmVSD.^4^ In this specific case, the girl had a 2.7 mm ventricular septal defect located behind the tricuspid septal leaflet. A double-disk symmetrical concentric pmVSD occluder (5 mm, LifeTech Scientific, Shenzhen, China) was used for closure, positioned just beneath the non-coronary aortic sinus (*Figure 3A*). The occluder consisted of a 0.127 mm Nitinol wire mesh with fabric inside. During RFCA, HB potentials were recorded at the margin of the occluder (*Figure 1G* and *H*). To protect the HPS, it is advisable to select targets from a relatively distant position. In hindsight, it would have been more prudent to thoroughly map all possible PVC origin locations, select the best target, and then proceed with the ablation.

It has been previously reported that radiofrequency energy can be enhanced by metallic implanted devices, resulting in an uncontrolled extent of injury.^5^ The presence of junctional beats and RBBB in the present case suggests injury of the HPS area. To our knowledge, the HPS was not located within the ablation zone. A plausible explanation may be that the metal device enhances conductive heat for energy delivery and extends the lesion area, resulting in injury to the HPS. Thus, it is important to carefully select the target and ablation energy. In this case, we chose to ablate at LVOT to prevent further damage to HPS. A similar strategy was previously proposed for fascicle PVC ablation, where ablation at the RCC was successful and safer in tackling PVCs originating from the proximal left anterior fascicles.^6^

Thus, it is important to thoroughly investigate the potential impact of metallic devices implanted within heart chambers on ablation. Further research is needed to establish a general consensus on how to address these issues.

In conclusion, this is a rare case of RBBB and junctional beats recorded during RFCA for outflow tract PVC in a patient with a history pmVSD and metallic occluder implantation. It highlights that RFCA energy delivery may be adversely affected by metallic occluders, although the underlying mechanism is unclear. This cautions electrophysiologists to thoroughly create an electroanatomic and activation map before beginning ablation, especially in complicated cases. Ablation at a relatively remote position is feasible for patients with metallic implants and congenital structural anomalies of the heart.

## Data Availability

The data underlying this article are available in the article.

## Lead author biography

Dr Weidong Lin graduated from the Guangdong Cardiovascular Institute in 2019. He is currently working at Guangdong Provincial People's Hospital, Department of Cardiovascular Medicine. He specializes in Catheter Ablation of Cardiac Arrhythmias.

**Consent:** The authors confirm that written consent for submission and publication of this case report including images and associated text has been obtained from the patient in line with COPE guidance.

**Funding:** This work was supported by the Zhongnanshan Medical Foundation of Guangdong Province (grant number no. 202151) and the Guangdong Special Funds for Science and Technology Innovation Strategy, China (stability support for scientific research institutions affiliated to Guangdong Province-GDCI 2021) (grant number KJ022021050).
